# COVID-19: scientific reasoning, pragmatism and emotional bias

**DOI:** 10.1186/s13613-020-00756-7

**Published:** 2020-10-12

**Authors:** Luciano Gattinoni, John J. Marini, Davide Chiumello, Mattia Busana, Luigi Camporota

**Affiliations:** 1 Department of Anesthesiology, Emergency and Intensive Care Medicine, University of Göttingen, Göttingen, Germany; 2 Pulmonary and Critical Care Medicine, Regions Hospital and University of Minnesota, St. Paul, MN USA; 3 Department of Anesthesia and Intensive Care, ASST Santi Paolo e Carlo, San Paolo University Hospital, Milan, Italy; 4 Department of Adult Critical Care, Guy’s and St Thomas’ NHS Foundation Trust, King’s Health Partners, and Division of Asthma, Allergy and Lung Biology, King’s College London, London, UK

Dear Editor,

We thank Dr. Tobin et al. for their comments [1] in response to our letter [2].

At this time of uncertainty, clinicians turn to experts and opinion leaders for advice on how to best manage a patient afflicted by a new and complex disease which affects primarily—but not exclusively—the respiratory system. Under the strains of pandemic practice, everyone is trying hard; clinicians must strike a sensitive and difficult balance in managing a relentless caseload with the limited (if not inadequate) resources at their disposal.

In the midst of early applause by the general public and intense scrutiny by healthcare systems and governments—it is clear that there has been wide variability in the provision of care for patients of similar severity affected by COVID-19. When all answers are in, is quite possible that some of the initial applause will turn eventually into fault-finding and condemnation.

In this context, it is very well to “sit on the fence” and from there launch darts of judgment at those who are trying to express a particular view, as long as these contestations help clinicians in their decision-making.

Yet, it seems to us that most of the arguments, counter-arguments, attempts to correct, disparage and set-the-record-straight expressed by Tobin et al. [1] are aimed, not so much at the worth or fault of the arguments per se, but at their proponents—namely, us. This impression stems from the observation that among the avalanche of articles published since January 2020, which contain a wide spectrum of opinions (as should be expected when the evidence is patchy and apparently inconsistent), none beside our own have received such an exacting response. Leaving aside these reflections, other considerations need to be brought to the fore: the quibbling and partial nature of the objections raised.

When trying to piece together the pathophysiology of this unfamiliar entity, to this point we have had to rely on fragmentary evidence, logic and scientific intuition. It is not difficult to imagine that to the ideas and examples we report in our manuscripts, Dr Tobin—or anyone else—will be able to find exceptions and contradicting evidence. The scientific literature is full of such examples. Anyone can do the same—if they simply wish to abrogate a point of view.

All intensivists know with certainty that invasive mechanical ventilation may be life-saving. No one proposes otherwise. Yet, we should not be criticized when we suggest that Tobin and co-authors had expressed views that premature intubation in this COVID context is “fatal”. For example, recently in the American Journal of Respiratory and Critical Care Medicine, we can find: *“the surest way to increase COVID*-*19 mortality is the liberal use of intubation and mechanical ventilation”* [3]. Clearly, the meaning and interpretation of the word “liberal” in this context is wide-ranging. However, the contention that mechanical ventilation is *“the surest way to increase COVID*-*19 mortality”* – is not supported by clinical data and therefore, once again such a statement is yet to be proved or disproved. Our detractors failed to note that we had focused our recommendations for early intervention on those patients who continue (or intensify) vigorously labored breathing with less invasive therapy.

Another example is provided when Tobin et al. say *“Patients with acute severe asthma develop large pleural pressure swings, yet autopsy studies in patients dying because of status asthmaticus are remarkable for the absence of pulmonary edema”* [1]. It is unfortunate that in the study quoted, the main histological characteristics—as the title of that paper suggests, “… reference to changes in the BRONCHIAL mucosa” [4]. Indeed, almost 2 decades later another uncited paper from a prominent journal reported edematous lungs in children with severe asthma [5]. It seems, therefore, that the strong breathing efforts of asthma as well as those during upper airways obstruction promote “negative pressure pulmonary oedema”. Similar hydrostatic forces are at work during labored breathing in the fluid-permeable lungs of COVID-19.

We agree that at present, worsening of a chest X-ray cannot be linked directly to P-SILI [1]; however, results of the recently published study by Tonelli [6] is mechanistically consistent with the hypothesis we have been trying to advance [2]. The tidal volumes and pressures during NIV are too variable and too influenced by the integrity of the interface seal to definitively support or disprove P-SILI. Ethics dictate that there can be no ‘*experimentum crucis*’ of deliberate iatrogenic injury, as seems to be requested from us by our critics.

We do not wish to maintain ongoing arguments with Tobin et al. [1]—who we respect as clinicians and scientists who have greatly contributed to the advancement of critical care. Nonetheless, readers do need to objectively consider the merit of such arguments and our response to them. By doing so, hopefully they may reconcile those that square best with their own clinical experience until these important management issues are definitively resolved.

Surely, we are entitled to put forward a hypothesis to the scientific community without meeting the same unremitting finger wagging response in a time of uncertainty that demands that we choose the most logical approach to clinical problems we cannot ignore. We are left with the same conclusion: to prove and disprove something is the basis of scientific progress. It is possible, then, that future data will disprove the non-existence of spontaneously induced lung injury or prove the tragic consequences of ignoring it.

## Data Availability

Not applicable.
